# Construction of recombinant fluorescent LSDV for high-throughput screening of antiviral drugs

**DOI:** 10.1186/s13567-024-01281-2

**Published:** 2024-03-16

**Authors:** Jingyu Wang, Jinzhao Ji, Yongcheng Zhong, Wenxin Meng, Shaobin Wan, Xiaoqing Ding, Zihan Chen, Weiyong Wu, Kun Jia, Shoujun Li

**Affiliations:** 1 College of Veterinary Medicine, South China Agricultural University, Guangzhou, China; 2 Guangdong Technological Engineering Research Center for Pet, Guangzhou, China; 3 Agriculture and Rural Affairs Bureau of Luocheng Mulao Autonomous County, Guangxi, China

**Keywords:** Lumby skin disease virus, recombinant virus, antiviral drugs, theaflavin

## Abstract

Lumpy skin disease virus (LSDV) infection is a major socio-economic issue that seriously threatens the global cattle-farming industry. Here, a recombinant virus LSDV-ΔTK/EGFP, expressing enhanced green fluorescent protein (EGFP), was constructed with a homologous recombination system and applied to the high-throughput screening of antiviral drugs. LSDV-ΔTK/EGFP replicates in various kidney cell lines, consistent with wild-type LSDV. The cytopathic effect, viral particle morphology, and growth performance of LSDV-ΔTK/EGFP are consistent with those of wild-type LSDV. High-throughput screening allowed to identify several molecules that inhibit LSDV-ΔTK/EGFP replication. The strong inhibitory effect of theaflavin on LSDV was identified when 100 antiviral drugs were screened in vitro. An infection time analysis showed that theaflavin plays a role in the entry of LSDV into cells and in subsequent viral replication stages. The development of this recombinant virus will contribute to the development of LSDV-directed antiviral drugs and the study of viral replication and mechanisms of action.

**Supplementary Information:**

The online version contains supplementary material available at 10.1186/s13567-024-01281-2.

## Introduction

Lumpy skin disease (LSD) is an acute or subacute infectious disease caused in cattle by lumpy skin disease virus (LSDV), and is characterized by fever and widespread nodules or ulcers on the skin or internal organs. LSDV affects all ages and breeds of cattle, with cows and calves at their peak lactation stage the most susceptible to infection. LSDV has a strong interspecific barrier and only infects other ruminants, such as sheep and camels [1]. The incidence rate of LSDV varies greatly, even on different farms in the same epidemic area, and is usually between 2 and 45%. The mortality rate is low. However, this disease can significantly reduce milk production in cows and induce weight loss in beef cattle, and can affect leather production, resulting in serious economic losses [2]. It is listed as a legally reportable disease by the World Organization for Animal Health (OIE).

LSDV is a orthopxvirus, of approximately 270 nm × 290 nm, with only one serotype and no hemagglutination activity [3]. LSDV proliferates in various cell types, including Madin–Darby bovine kidney (MDBK) cells, bovine testicular cells, sheep embryonic kidney cells, lamb testicular cells, and African green monkey kidney (Vero) cells [4]. The total length of the LSDV genome is 145–156 kbp, and it consists of two central coding regions defined by the same 2.4-kbp inverted terminal repeat (ITR) regions [5]. The A+T content of the genome is 73%, and it includes 156 putative genes [5]. LSDV contains most of the conserved poxvirus genes related to viral replication, including at least 26 genes involved in RNA polymerase subunits, mRNA transcription initiation, extension, and termination, as well as enzymes that must be modified or processed after viral mRNA transcription.

LSDV has recently spread widely in Asia. Since the first confirmed outbreak of LSD in Xinjiang, China in August 2019 [6], it has emerged in countries such as Vietnam [7], Myanmar [8], Thailand [9], Cambodia [10], Malaysia, Laos, Indonesia, Singapore, India [11], Bangladesh [12], Bhutan [13], Pakistan [14], Nepal [15], and Sri Lanka [13]. Therefore, controlling LSDV infection has become a priority in the cattle production industry. During the current LSDV pandemic, inactivated vaccines have been commonly used for emergency immunization, whereas antibiotics are used to treat secondary bacterial infections in cattle. However, this strategy is increasingly questioned because it can lead to the emergence of drug-resistant bacteria and inactivated vaccines induce immunity of only limited duration, which makes them very costly [16]. Therefore, effective treatment methods are still required that limit viral replication and reduce the damage caused by the virus. Clearly, appropriate cheap and effective traditional Chinese medicinal monomers could greatly reduce the economic impact of LSDV.

The homologous recombination of DNA is a core technology in molecular biology, which allows the introduction of foreign genes into viral genomes [17]. This method can simultaneously delete virulence genes, weakening the virus. In this way, attenuated vaccines can be prepared. In a previous study, a partial thymidine kinase (TK) gene of pseudorabies virus (PRV) was used as the homologous recombination sequence, and the green fluorescent protein (EGFP) gene was used as a screening marker to construct a recombinant PRV carrying the EGFP gene. After the blind transmission of the virus into Vero cells for 18 generations, the green fluorescent protein was still expressed, and the virus could be used for drug screening or vaccine preparation [18]. In the present study, we developed a recombinant LSDV expressing EGFP-labeled protein using this homologous recombination technology. We describe the characteristics of this virus and the optimization of its application in 96- or 48-well plates. We validated our new construct by screening a library of natural Chinese medicinal monomers (100 compounds) for antiviral compounds. With a high-throughput screening system, we identified emodin, aloe emodin, theaflavin, 4-ethylphenol, and tulipalin as having anti-LSDV-infection effects in vitro. The optimal times for the addition of theaflacin was determined to identify the stage/s at which they inhibit viral infection.

## Materials and methods

### Cells and virus

Madin–Darby bovine kidney (MDBK) cells, Vero cells, Crandel feline kidney (CRFK) cells, porcine kidney epithelial (PK15) cells, and Madin–Darby canine kidney (MDCK) cells were propagated in Dulbecco’s modified Eagle’s medium (DMEM; Gibco, New York, USA) supplemented with 10% fetal bovine serum (FBS; Gibco, New York, USA), 100 units/mL penicillin, and 100 μg/mL streptomycin. All mammalian cells were grown at 37 °C under a 5% CO_2_ atmosphere.

LSDV/MZGD/2020 (OP985536.1) was isolated from domestic cattle in Guangdong, China [6], and is maintained in our laboratory. The wild-type LSDV (LSDV-WT) stock was propagated in MDBK cells in DMEM containing 2% FBS, and aliquots were stored at −80 °C. LSDV-ΔTK/EGFP was generated through the transfection of Vero cells with the plasmids described below. All work with infectious virus was conducted in a biosafety level-2 (BSL-2) laboratory.

### Plasmid construction

**Figure 1:**
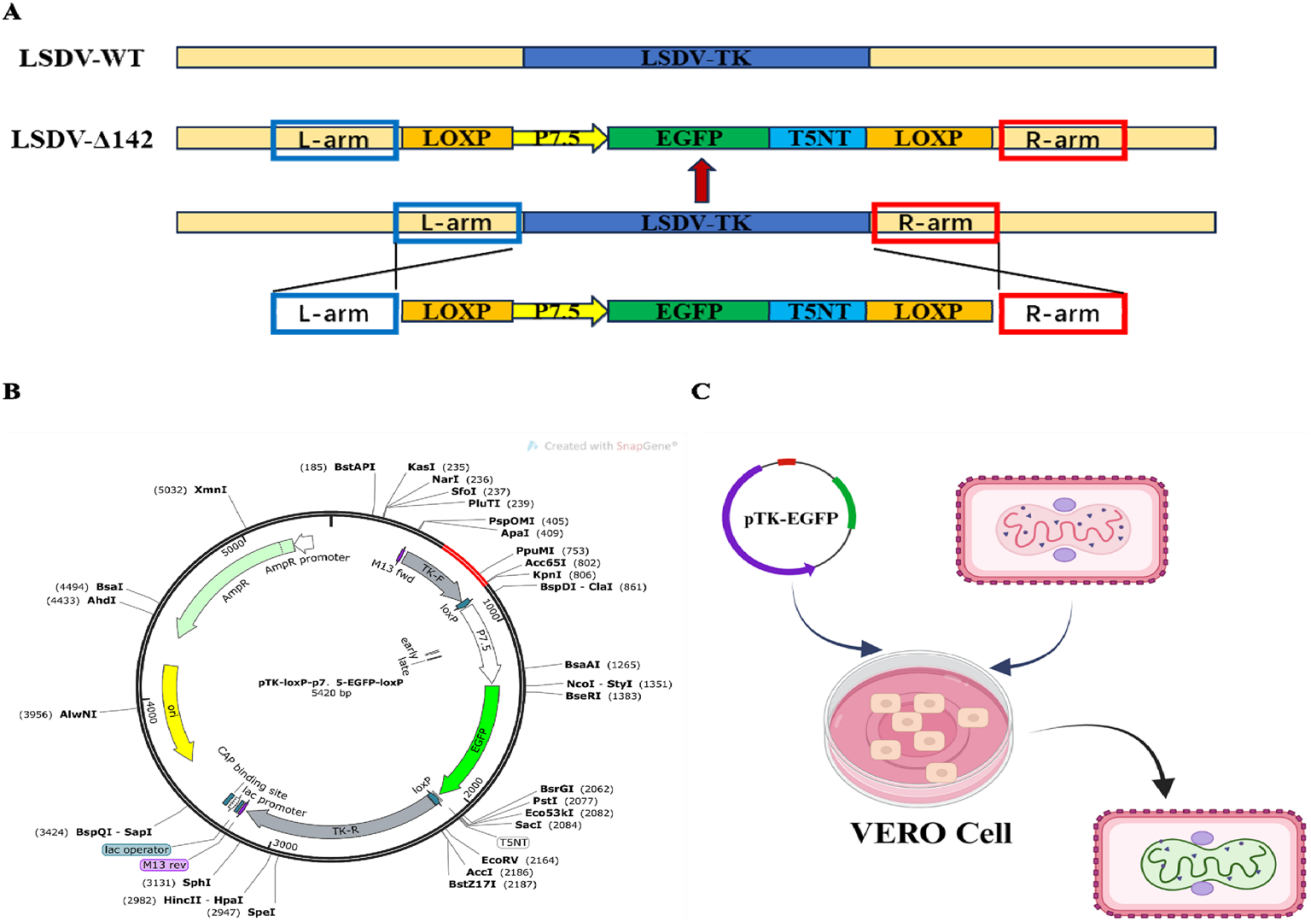
**Structure diagram and transfection strategy of the recombinant plasmids to rescue the recombinant LSDV.** **A** Schematic showing the genomes of wild-type and recombinant LSDV strains. **B** Schematic diagram of recombinant transfer plasmid construction, named pTK-loxp-p7.5-EGFP-loxp. **C** Transfection strategy for LSDV-WT and pTK-loxp-p7.5-EGFP-loxp.

Transfection and infection procedures were used to create LSDV with a TK gene deletion generated by homologous recombination between LSDV-WT and recombination transfer vectors (Figure 1A). Based on the entire genomic sequence of LSDV-WT, multiple primers were designed with the Primer Premier 5 software (Premier, Canada): TK-LTYB-F (5′-ATGGACTATGGATATATACATTTAATT 3′)/TK-LTYB-R (5′-TGGCCTTCATCTATACCTATA-3′) (265 bp) and TK-RTYB-F (5′-ATTGTATCTTTTTCTGAAAATAT-3′)/TK-RTYB-R (5′-TTATTCTAAAAAATAACATTTCCTAC-3′) (269-bp fragment). The Magen MagPure Viral RNA/DNA Kit (Magen, Guangzhou, China) was used to extract the viral genome. We used Takara PrimeSTAR® Max DNA Polymerase (Takara, Japan) to amplify the left and right homologous arms of the TK gene and to sequence the PCR product for confirmation. We cloned the TK homologous arms and the EGFP gene into the pMD18-T Simple Vector (Takara, Japan) containing the LoxP (Cre/LoxP recombinase system), the poxvirus promoter P7.5, and the pox terminator T5NT, generating the plasmid pTK-loxPp7.5-EGFP (Figure 1B). The loxP site is specifically recognized by the Cre recombinase protein, causing the deletion of the sequence between two LoxP sites. All the constructs were confirmed with DNA sequencing.

### Virus rescue and purification

Lipofectamine™ 3000 Transfection Reagent (Thermo Fisher Scientific) was used to transfect Vero cells with 2 μg of the recombinant transfer vectors. The cells were then infected with LSDV-WT at a multiplicity of infection (MOI) of 1 at 8 h post-transfection (hpt). The cells expressing fluorescence were picked from the Vero monolayers under a fluorescence microscope (Figure 1C). The harvested cells were freeze–thawed and the virus transferred into fresh MDBK cells. Plaques expressing fluorescence were picked from the MDBK monolayers under a fluorescence microscope. After 8–10 rounds of plaque screening and further dilution purification, the virus containing the gene deletion was purified to homogeneity. Using this construction method, we generated TK-gene-insert LSDV, designated LSDV-ΔTK/EGFP.

Screening fluorescent cells. A capillary glass tube was heated and pulled to a diameter of 2–40 μm. The cell culture plate was placed under a fluorescence microscope, and excited green or red cells were drawn into the tube. The cells were transferred into a sterile 1.5 mL EP tube containing 200 μL of DMEM and stored at −80 °C.

### Viral growth kinetics

To compare the differences in the replication of LSDV-WT and LSDV-ΔTK/EGFP, the growth kinetics of the two viruses were examined. A six-well plate was inoculated with about 1 × 10^5^ MDBK cells. After incubation overnight, the cells were infected with LSDV-WT or LSDV-ΔTK/EGFP at an MOI of 1. After incubation for 2 h, the supernatant was collected and stored at −80 °C. The viral titer was measured as the median tissue culture infective dose (TCID_50_). After LSDV-ΔTK/EGFP infection, the expression of the EGFP gene was observed under a fluorescence microscope at 100× magnification. Only LSDV formed plaque-like lesions on infected cells, so we identified fluorescent green plaques overlapping cell lesions to quantify the EGFP signal and compared it with the bright field.

### Stability of the LSDV-ΔTK/EGFP virus in cell culture

To analyze the stability of the *EGFP* reporter gene during viral passage, LSDV-ΔTK/EGFP was serially passaged 20 times on MDBK cells. In each generation, 200 μL of viral solution was collected and the viral genome extracted. The Primer5 software was used to design the detection primers used to amplify the LSDV-ΔTK/EGFP insertion fragment: TK-loxp-F (5′-ATGGACTATGGATATATACATTTAATT-3′)/TK-loxp-R (5′-TTATTCTAAAAAATAACATTTCCTAC-3′). The genome detection was amplified between the left and right homologous arms to detect the EGFP fragment and the PCR amplification products were sequenced.

### Transmission electronic microscopy

A six-well plate was inoculated with Vero cells at the appropriate density. The Vero cells were infected with LSDV-WT or LSDV-ΔTK/EGFP at an MOI of 1 for 72 h. The cells were collected and fixed with precooled electron-microscope fixative purchased from Wuhan Servicebio Technology (Wuhan, China). For the subsequent experiment, we handed control over Wuhan Servicebio Technology (Wuhan, China).

### Cell sensibility to LSDV-WT and LSDV-ΔTK/EGFP

Twenty-four-well plates were seeded with cells from different sources, including Vero, PK15, MDBK, MDCK, and CRFK cells, at 1 × 10^5^ cells per well. After incubation overnight, the cells were infected with LSDV-WT or LSDV-ΔTK/EGFP at an MOI of 1. After incubation at 37 °C for 2 h, the viral diluent was discarded, fresh cell culture medium was added, and the cells were cultured at 37 °C. The cells inoculated with LSDV-ΔTK/EGFP were observed and photographed every day (from day 0 to day 5) to record the viral infection. The cells infected with LSDV-WT were fixed at 2 days post-infection (dpi), and subjected to an immunofluorescence assay with rabbit anti-ORF142 antibody (Save in our laboratory).

### Compound screening

A library of natural Chinese medicinal monomers (100 compounds) for antiviral compounds (Additional file 1) was purchased from Top Science. The compounds were stored as 10 mM stock solutions in dimethyl sulfoxide (DMSO) or ddH2O at −80 °C until use. MDBK cells were dissociated and seeded in 48-well plates at a density of 5 × 10^4^ cells per well. After incubation overnight, the cell monolayers were infected with LSDV-ΔTK/EGFP at an MOI of 0.1 for 1 h. The compounds were prepared to final concentrations of 10 μM with DMEM containing 2% FBS and 0.1% DMSO. ddH2O was used as the negative control. After incubation for 72 h, the fluorescent signal was detected in all the wells with fluorescence microscopy and the number of cells expressing EGFP was calculated.

### Immunofluorescence assay

Vero, PK15, MDBK, MDCK, and CRFK cells in 10 mm confocal dishes were infected with LSDV-WT at an MOI of 1 for 72 h. The cells were then washed three times with phosphate-buffered saline (PBS), fixed with 4% paraformaldehyde for 15 min at 4 °C, permeabilized with 0.02% Triton X-100 in PBS for 10 min, and blocked with PBS containing 5% skimmed milk powder for 60 min at 37 °C. After three quick washes, the cells were incubated with a rabbit anti-ORF142 antibody (Save in our laboratory) overnight at 4 °C, washed three times with PBS, and then incubated with an Alexa Flour 594 goat anti-rabbit IgG (H+L), secondary antibody (Cell Signaling Technology) for 2 h at 37 °C. Finally, the cells were washed thoroughly three times with PBS and incubated with 4′,6-diamidino-2-phenylindole (DAPI) staining solution for 10 min. The cells were viewed under a fluorescence microscope (Leica, Frankfurt, Germany).

### Cytotoxicity assays

The cytotoxicity of the compounds to MDBK cells was evaluated with a Cell Counting Kit-8 (CCK-8, Beyotime, Shanghai, China) assay. Confluent cells in 96-well cell culture plates (seeding density of 2 × 10^4^ cells/well) were incubated with different concentrations of compounds for 72 h at 37 °C under 5% CO_2_. The medium was removed after incubation, and the cells were washed with sterile PBS before CCK-8 solution was added (Vazyme, Nanjing, China). Finally, the cells were incubated at 37 °C for another 2 h and subjected to colorimetric measurements with a microplate reader at 450 nm.

### Quantitative PCR (qPCR)

The LSDV genomic DNA was extracted from cell supernatants and lysates with the Kingfisher Flex System (Thermo Fisher Scientific), according to the manufacturer’s instructions. ORF142 of LSDV was quantitatively determined with the Light-Cycler® 480 Real-Time PCR System (Roche, Basel, Switzerland). Amplification was conducted in a 20 µL reaction mixture containing 2 µL of genomic DNA, 0.4 µL of forward primer (5′-GCGAAGAACGTGAACTAAA-3′), 0.4 µL reverse primer (5′-CTTCTGCATTCAACCCATC-3′), 10 µL of ChamQ Universal SYBR, and 7.2 µL of sterile water. The qPCR thermal cycling conditions were 95 °C for 3 min, followed by 40 cycles of 95 °C for 15 s and 55 °C for 10 s, and a final step at 60 °C for 30 s. The viral genome copies in the samples were determined as the number of LSDV ORF142 genes and calculated with a standard curve.

### Western blotting

MDBK cells infected or not infected with LSDV-WT were washed three times with cold PBS at specific time points and then harvested with lysis buffer containing 1% protease inhibitors (Beyotime). The protein concentration was determined with a BCA Protein Assay Kit (Beyotime). The cell extracts were resolved with sodium dodecyl sulfate polyacrylamide gel electrophoresis and transferred to polyvinylidene difluoride membranes (Beyotime). Total ORF142 protein was detected with a rabbit anti-ORF142 antibody (stored in our laboratory), with rabbit anti-β-actin (Proteintech, Wuhan, China) as the loading control.

### Statistical analysis

Data are presented as means ± standard errors of the mean of at least three replicates. T tests were performed with GraphPad Prism 5.0 (GraphPad Software Inc., CA, USA). The abbreviation “ns” indicates no significant difference (*P* > 0.05); * indicates a significant difference (*P* < 0.05); ** indicates a highly significant difference (*P* < 0.01); and *** indicates an extremely significant difference (*P* < 0.001).

## Results

### The rescue and characterization of LSDV-ΔTK/EGFP

**Figure 2:**
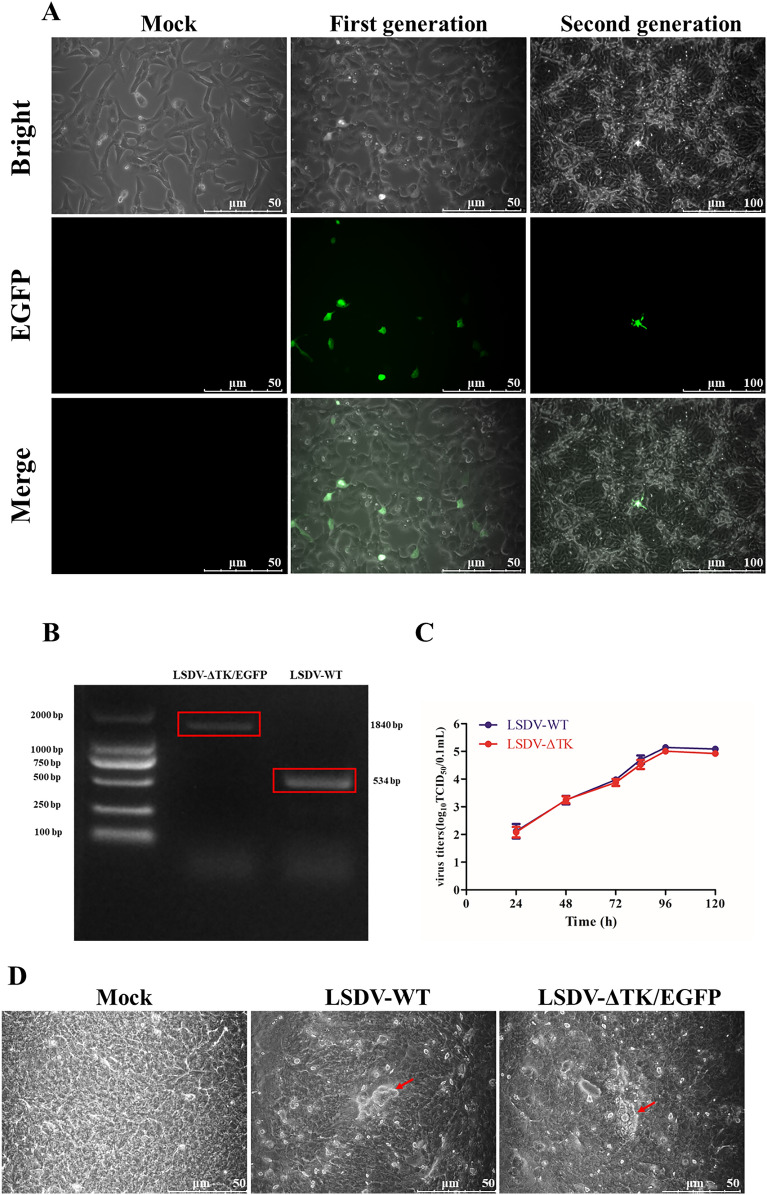
**Characterization of LSDV-WT and LSDV-ΔTK/EGFP.** **A** The proliferation of recombinant viruses in VERO cells was observed in mock, first generation, and second generation. **B** The RT-PCR detection of the EGFP gene in MDBK cells infection with LSDV-WT or LSDV-ΔTK/EGFP. **C** Growth kinetics curves of LSDV-WT and LSDV-ΔTK/EGFP in the MDBK cells at MOI = 1. **D** The morphology of CPE in MDBK cells of LSDV-ΔTK/EGFP and LSDV-WT on 4 dpi.

The plasmid constructed to rescue LSDV-ΔTK/EGFP was confirmed with DNA sequencing. After the transfection of the pTK-loxP-EGFP plasmid encoding LSDV-ΔTK/EGFP, green fluorescence appeared in Vero cells at 24 hpi (Figure 2A), confirming that the p7.5 promoter in the transfer vector correctly activated the expression of the *EGFP* gene. Monoclonal cell selection technology was used to select cells that stimulated a single green fluorescence and continued to infect MDBK cells. Single viral plaques were selected (Figure 2A) based on green fluorescence, for the next round of screening. After multiple rounds of purification, only LSDV expressing EGFP was isolated. The recombinant virus was collected and its total DNA was extracted to detect the expression of EGFP with reverse transcription (RT)–PCR with primers TK-loxp-F/R, to confirm the identity of the virus. A fragment of approximately 534 bp (TK) was amplified from the parental strain LSDV/MZGD, whereas a fragment of 1840 bp (TK + EGFP) was amplified from the screened recombinant viral culture, confirming the insertion of the EGFP gene into the viral genome (Figure 2B). To investigate the differences in the growth performance of the WT and recombinant viruses, we determined the titers of the viruses in MDBK cells at different time points, and plotted their growth curves. The results showed that after infection with the same virus at the MOI, the production and replication patterns of the WT and recombinant viruses were similar (Figure 2C). In MDBK cells, the acne-like lesions formed by LSDV-ΔTK/EGFP were similar to those of the WT virus, showing cell shrinkage, brightening, clustering, and a stronger refractive index than uninfected cells, followed by cell contraction and aggregation to form nodules (Figure 2D). The EGFP signal showed the same trend as the viral titer in both LSDV strains.

These results indicate that the EGFP gene was successfully introduced into the TK gene of the virus. The growth performance of and pathological changes caused by the rescued recombinant virus were consistent with those of the WT virus. The findings also indicate that the TK gene deletion did not affect the replication ability of the virus.

### EGFP was stably expressed during LSDV-ΔTK/EGFP generation

**Figure 3:**
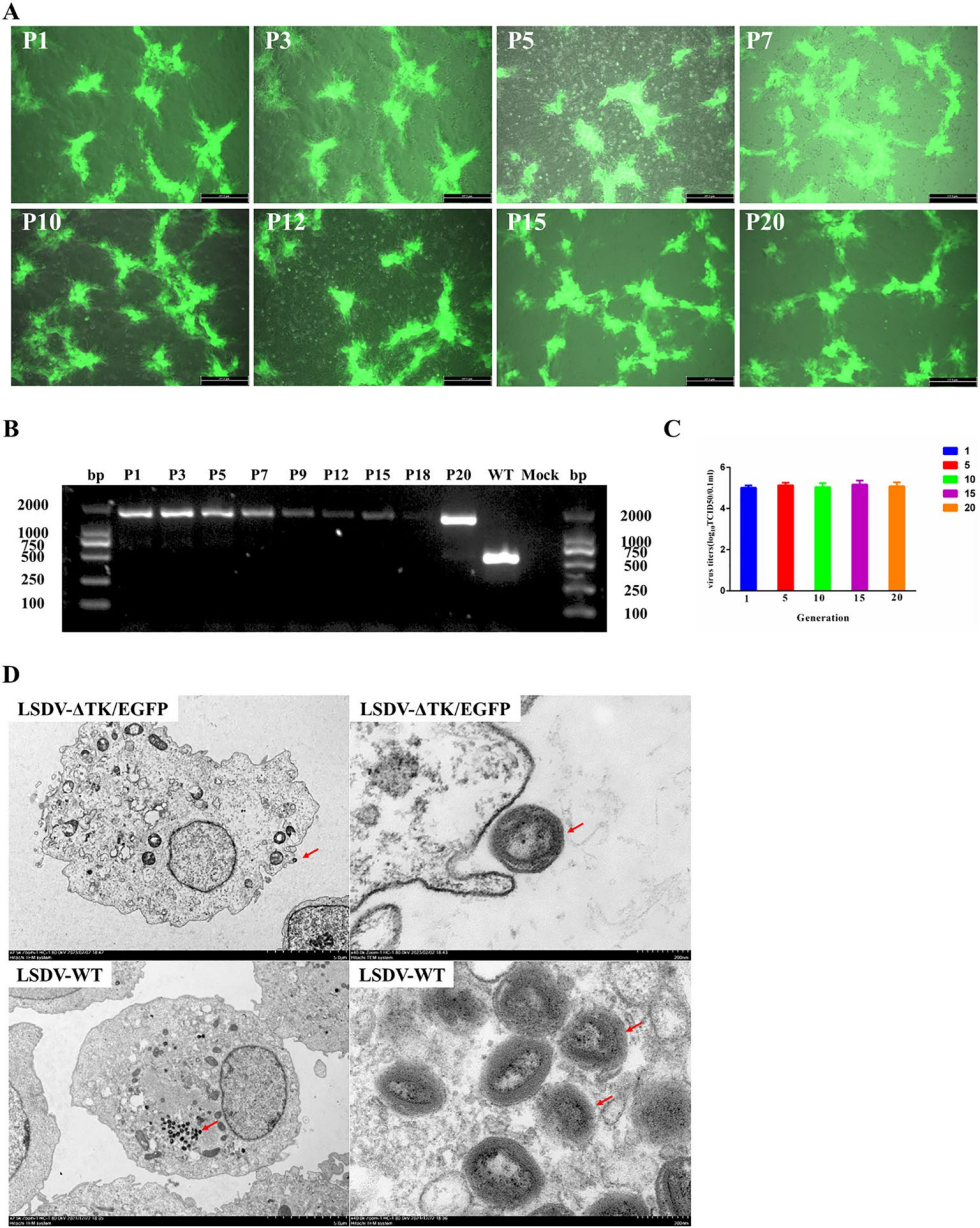
**The EGFP stability in LSDV-ΔTK/EGFP during passaging in MDBK cells.** **A** The EGFP expression in MDBK cells after infection with LSDV-ΔTK/EGFP of P1–P20. The expression of EGFP was detected under a fluorescent microscope at 96 hpi. **B** The RT-PCR detection of the TK gene in MDBK cells after infection with LSDV-ΔTK/EGFP of P1–P20 The resulting RT-PCR products were resolved by 1% agarose gel electrophoresis. **C** The LSDV-ΔTK/EGFP titer of P1, P5, P10, P15 and P20 generation.

To confirm the stability of LSDV-ΔTK/EGFP, the virus was serially passaged 10 times in MDBK cells. All the MDBK cells infected with LSDV-ΔTK/EGFP in passages P1–P20 showed strong green fluorescent signals, indicating the expression of *EGFP* gene was stable during passaging (Figure 3A). At each passage, the DNA of the infected cells was extracted and analyzed with RT–PCR to test the stability of the gene. Because a fragment of 1306 bp was introduced into the TK gene and 534 bp was introduced into the TK gene, an 1840-bp band was detected in LSDV-ΔTK/EGFP in passages P1–P20, whereas a band of only 534 bp was detected in LSDV-WT (Figure 3B). In P1–P20, LSDV-ΔTK/EGFP displayed this specific band, which was confirmed with DNA sequencing, and no sequence deletion, further suggesting the stability of EGFP in LSDV-ΔTK/EGFP in MDBK cells. The viral titers of LSDV-ΔTK/EGFP at P1, P5, P10, P15, and P20 were also determined and all of them were stable at concentrations of > 10^5^ TCID_50_/0.1 mL (Figure 3C).

To further investigate the morphological changes in LSDV-ΔTK/EGFP, we made ultrathin sections of infected cells for electron microscopic observation. The LSDV-ΔTK/EGFP and LSDV-WT viral particles underwent no morphological changes, either in the size of the viral particle (approximately 230 nm × 280 nm), the outer layer of the capsule, or the central chromatin (Figure 3D).

### LSDV-ΔTK/EGFP and LSDV-WT infect cell lines from different hosts

**Figure 4:**
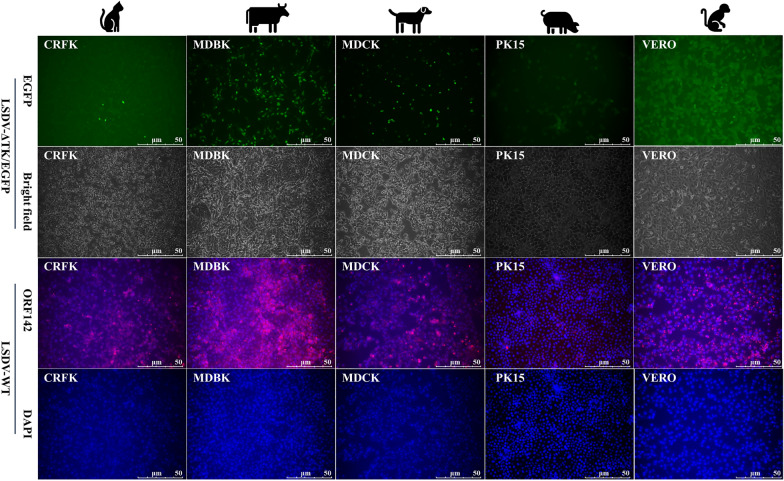
**The infection of LSDV-ΔTK/EGFP and LSDV-WT in cell lines derived from cat, cattle, dog, pig and monkey.** **A** the EGFP (green) expression in different cell lines infected LSDV-ΔTK/EGFP at an MOI = 1 on 4 dpi. **B** IFA of ORF142 protein (red) and DAPI (blue) in different cell lines on 4 dpi after LSDV-WT infected at MOI = 1. The experiment was repeated 3 times.

To investigate the sensitivity of to different host cells for LSDV, we infected five different cell lines separately with LSDV-ΔTK/EGFP and LSDV-WT, and detected the proliferation of the two viruses in the cells of different species. All five cell lines were infected, but with different sensitivities. The renal epithelial cells of dogs and cats were highly sensitive to both LSDV-ΔTK/EGFP and LSDV-WT, indicating that LSDV may be transmitted between dogs and cats. However, PK15 (pig) cells showed lower sensitivity to LSDV-ΔTK/EGFP and LSDV-WT than dog and cat cells, whereas Vero (monkey) showed higher sensitivity to LSDV-ΔTK/EGFP and LSDV-WT. Furthermore, compared with dog, cat, pig, and monkey cells, MDBK cells showed extremely high sensitivity to LSDV-ΔTK/EGFP and LSDV-WT, which may be consistent with the fact that LSDV was originally isolated from cattle (Figure 4).

### Screening a library of natural monomeric small-molecule compounds of traditional Chinese medicine for anti-LSDV activity

**Figure 5:**
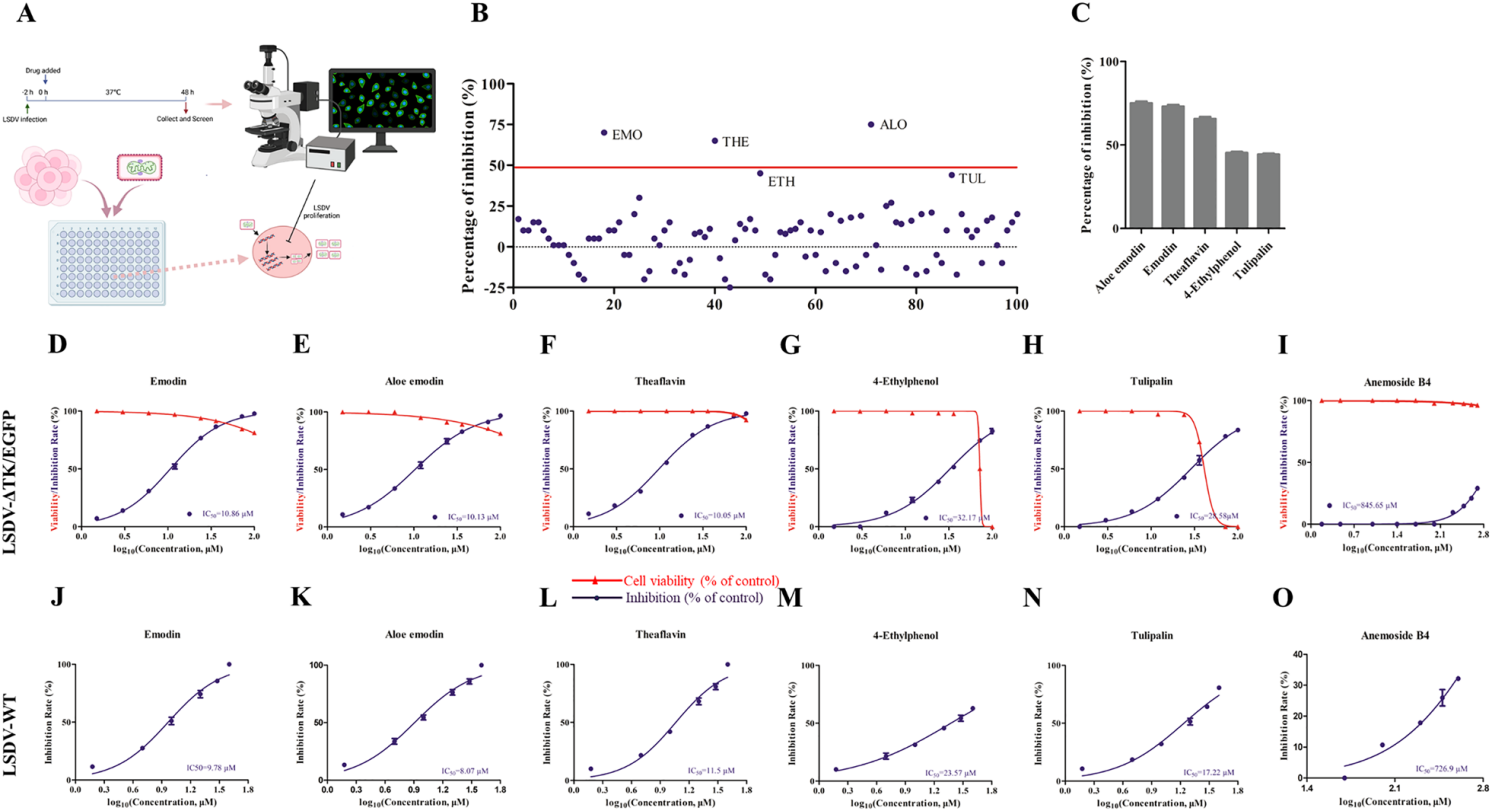
**Screening, dose–response curves and toxicity of antiviral compounds against LSDV using the traditional Chinese medicine monomer library.** **A** Assay scheme: MDBK cells were treated with compounds and LSDV-ΔTK/EGFP (MOI = 1) and incubated for 72 h. The number of cells that stimulate green fluorescence is counted through a fluorescence microscope and calculated based on a reduction in the number of EGFP expression cells. **B** Screening of 100 traditional Chinese medicine for primary candidates that inhibit LSDV-ΔTK/EGFP infection. Each dot represents the percent influence achieved with each compound at a concentration of 10 μM, which was calculated compared to that of the DMSO-treated control group. **C** These compounds of exhibiting significant inhibitory effects were shown in the bar graph. **D**–**I** MDBK cells were treated with different concentrations of candidate compounds (100, 72, 36, 24, 12, 6, 3 and 1.5 μM) or (Anemoside B4, 500, 400, 300, 200, 100, 50, 25, 10, 3, 1 μM) and infected with LSDV-ΔTK/EGFP (MOI = 0.1). Data showed the ratio of inhibition in LSDV-ΔTK/EGFP-infected cells (blue) and the ratio of cell viability in uninfected cells (red). **J**–**O** MDBK cells were treated with different concentrations of candidate compounds (100,72, 36, 24, 12, 6, 3 and 1.5 μM) or (Anemoside B4, 500, 400, 300, 200, 100, 50, 25, 10, 3, 1 μM) and infected with LSDV-WT (MOI = 0.1) for 96 h. The culture supernatant and cell lysates were collected to determine virus titers by TCID_50_. Data showed the ratio of inhibition in LSDV-WT-infected cells (blue).

To identify agents that potentially act against LSDV, we selected the MDBK cells to screen this library of compounds. MDBK cells are the natural target cells of LSDV, and this cell type supports high levels of infection. We screened a library of 100 Chinese medicinal monomers that exert antiviral and anti-inflammatory effects. During the screening process, we used the fluorescence intensity of LSDV-ΔTK/EGFP to characterize viral replication. The inhibition rate of each drug was ultimately calculated as the number of cells that expressed green fluorescence (Figure 5A). Monomeric drugs with an inhibition rate on LSDV of approximately 50% were defined as the main candidate drugs. Emodin, aloe emodin, theaflavin, 4-ethylphenol, and tulipalin exerted good inhibitory effects on LSDV replication (Figures 5B, C).

Anemoside B4 is already used as an anti-inflammatory and antiviral agent. Therefore, we tested the five drugs with significant inhibitory effects in parallel with anemoside B4 in subsequent experiments. When we used LSDV-ΔTK/EGFP to evaluate the inhibitory effects of various drugs, emodin, aloe emodin, and theaflavin all inhibited LSDV replication with different levels of cytotoxicity (Figures 5D–F), with 50% inhibitory concentrations (IC50) of 10.86 μM, 10.13 μM, and 10.05 μM, respectively. We also calculated the TCID_50_ of LSDV-WT, and showed that emodin, aloe emodin, and theaflavin also inhibited LSDV replication with varying degrees of cytotoxicity (Figures 5J–L), with IC50 values of 9.78 μM. 8.07 μM, and 11.5 μM, respectively. Cell Counting Kit-8 (CCK-8) was used to measure cell viability, and the results indicated that the cytotoxicity of these three drugs was very low, and drug concentrations of < 40 μM allowed > 80% cell viability (Figures 5D–F). Disappointingly, the inhibitory effects of 4-ethylphenol and tulipalin were lower than those of emodin, aloe emodin, and theaflavin, with IC50 values of 32.17 μM and 28.58 μM, respectively (Figures 5G–H). The inhibitory effects of 4-ethylphenol and tulipalin on LSDV-WT were similar to those on LSDV-ΔTK/EGFP, with IC50 values of 23.57 μM and 17.22 μM, respectively (Figures 5M–N). The inhibitory efficiency of anemoside B4 was lowest, with an IC50 values of 845.65 μM for LSDV-ΔTK/EGFP (Figure 5I) and 726.9 μM for LSDV-WT (Figure 5O). Overall, these results indicate that the anti-LSDV activities of compounds can be evaluated rapidly with this *EGFP* reporter virus.

### Effects of theaflavin on viral titer and protein synthesis in MDBK cells

**Figure 6:**
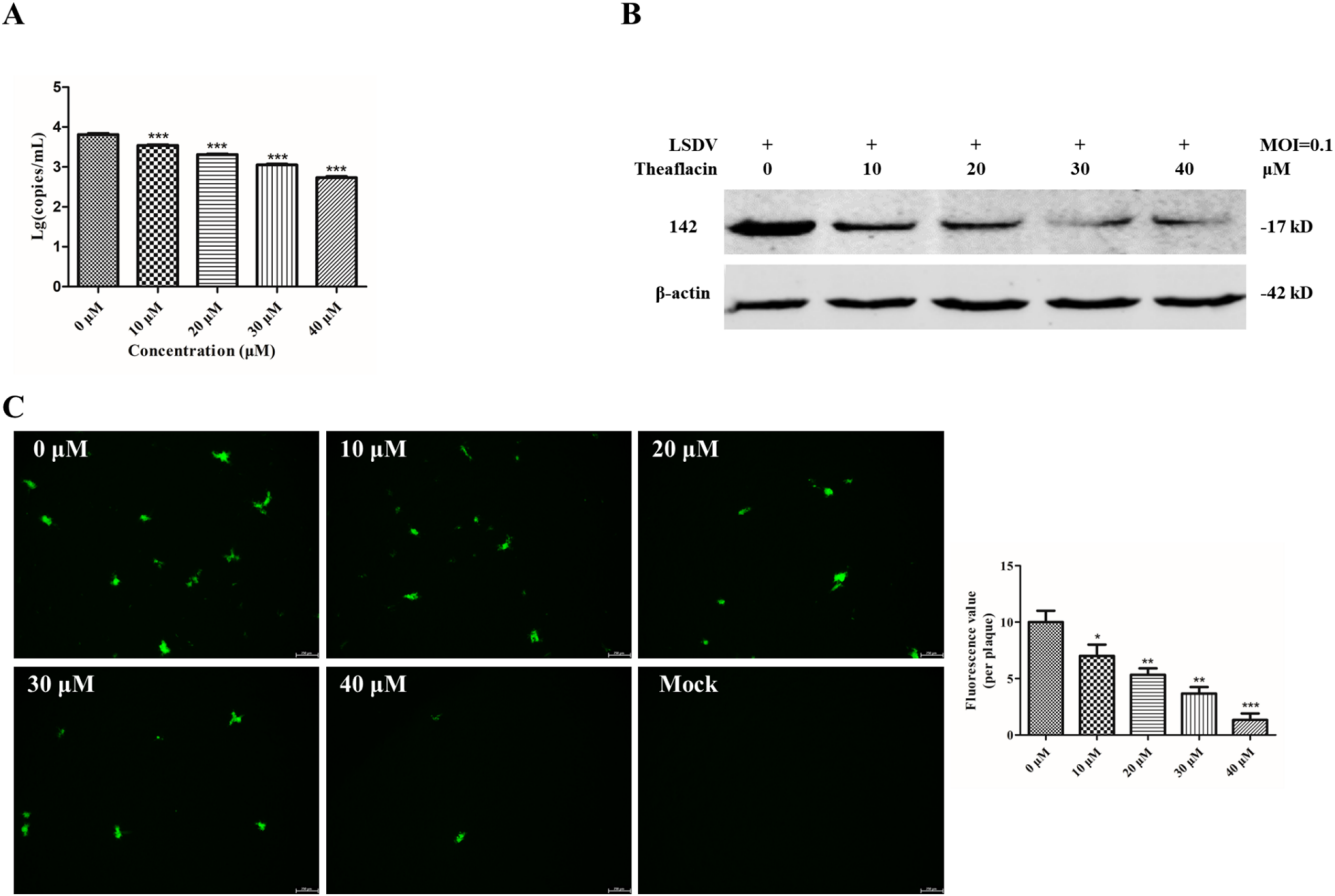
**Inhibitory effect of theaflavin on LSDV replication.** MDBK cells were treated with different concentrations (40, 30, 20, 10 and 0 μM) of theaflavin and infected with LSDV-ΔTK/EGFP or LSDV-WT at a MOI of 0.1. **A** Total DNA of the culture supernatant and cell lysates were co-collected at 72 hpi to determine virus genome copy number for LSDV-WT. **B** The cell lysates were collected at 48 hpi to detect the expression of LSDV ORF142 proteins by Western blot for LSDV-WT. **C** Fluorescent microscope was used to detect the expression of EGFP to determine the antiviral activity of theaflavin at different doses against LSDV in MDBK cells at 72 hpi for LSDV-ΔTK/EGFP. Data represented three independent experiments with three technical replicates (shown as mean ± SEM), and T-tests was performed. ns, not significant (*P* > 0.05); *, *P* < 0.05; **, *P* < 0.01; ***, *P* < 0.001.

To further evaluate the inhibitory activity of theaflavin on LSDV, we investigated the effect of this compound on LSDV-WT protein 142 and genome replication (RPO30 gene). An RT–qPCR assay showed that treatment with the compound at nontoxic concentrations (0, 10, 20, 30, and 40 μM) resulted in dose-dependent reductions in the number of viral genomes (Figure 6A). Using a Western blotting analysis, we evaluated the expression of the ORF142 protein in the presence of different concentrations of theaflavin. Treatment with various concentrations significantly inhibited the synthesis of ORF142 protein (Figure 6B). We simultaneously used LSDV-ΔTK/EGFP to evaluate the effect of theaflavin on EGFP expression. After treatment with theaflavin, the number of cells that expressed green fluorescence decreased significantly and dose-dependently compared with the control group (Figure 6C). Overall, these data indicate that theaflavin inhibits the replication of LSDV in target cells.

### Theaflavin inhibits LSDV infection during viral DNA replication without directly inactivating the virus

**Figure 7:**
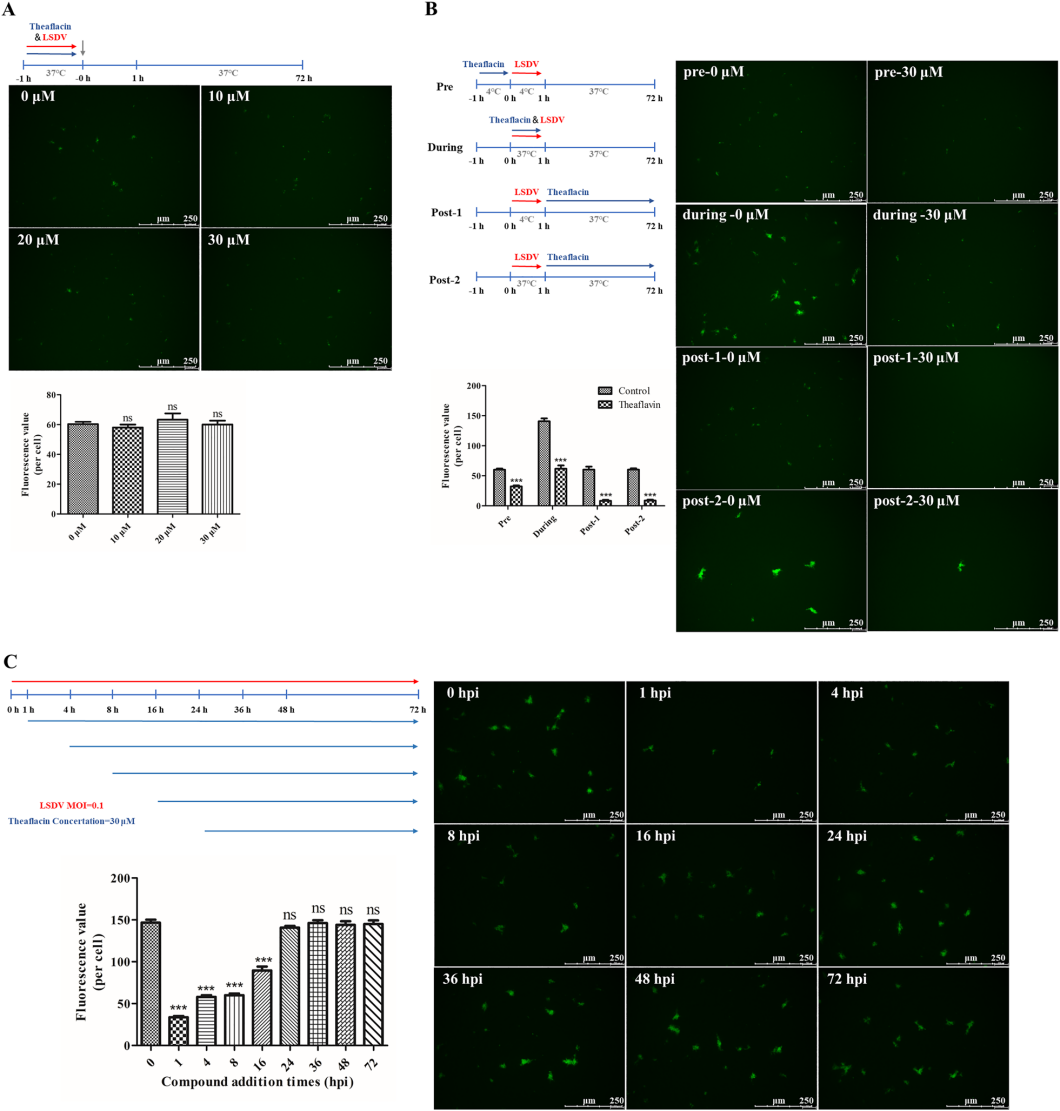
**Time-of-addition analysis of the antiviral activity of theaflavin.** **A** Schematic illustration of the virucidal assay (top). Theaflavin (10, 20 and 30 μM) and LSDV-ΔTK/EGFP (MOI = 0.1) were mixed and incubated at 37 °C for 1 h and then diluted 50-fold before adding to MDBK cells. The possible inactivating effect of theaflavin on the expression of EGFP was determined by fluorescent microscope assay at 72 hpi (middle) and the number of EGFP expressing cells is calculated (bottom). **B** Schematic illustration of the primary time-of-addition experiment (upper left). MDBK cells were treated with theaflavin (30 μM) and LSDV-ΔTK/EGFP (MOI = 0.1) at different times as indicated, and the possible inactivating effect of theaflavin on the expression of EGFP was determined by fluorescent microscope assay at 72 hpi (right) and the number of EGFP expressing cells is calculated (lower left). **C** Schematic illustration of the second time-of-addition experiment (upper left). MDBK cells were infected with LSDV-ΔTK/EGFP at an MOI of 0.1 and treated with theaflavin (30 μM) at different time points (1, 4, 8, 16, and 24 hpi). The possible inactivating effect of theaflavin on the expression of EGFP was determined by fluorescent microscope assay at 72 hpi (right) and the number of EGFP expressing cells is calculated (lower left). Data represented three independent experiments with three technical replicates (shown as mean ± SEM), and T-tests was performed. ns, not significant (*P* > 0.05), ***P < 0.001.

To investigate whether theaflavin exerts its antiviral effect by directly inactivating the virus, various concentrations (10, 20, and 30 μM) were incubated with LSDV-ΔTK/EGFP directly for 1 h at 37 °C. The mixture was then diluted 50-fold to noninhibitory concentrations (0.2, 0.4, and 0.6 μM) and added to MDBK cells for 72 h. The number of cells expressing EGFP protein was determined with fluorescence microscopy. As shown in Figure 7A, EGFP expression was not affected by preincubation with theaflavin, indicating that theaflavin does not inactivate the virus directly.

To determine the stage of the viral replication cycle targeted by theaflavin, we conducted a time-of-addition experiment in MDBK cells. Cells were infected with LSDV (MOI = 0.1) and then 30 μM theaflavin was added at different times during the experiment. The antiviral activity of theaflavin was observed in all stages of viral infection. Interestingly, the addition of theaflavin after virus incubation at 4 °C or 37 °C for 1 h had a significant inhibitory effect on LSDV replication (Figure 7B). These results indicate that theaflavin affects viral replication in the post-entry stage. To further investigate the stage of infection in which theaflavin acts, we measured the effect of adding theaflavin at different time points on LSDV replication, and observed the expression of EGFP protein in LSDV-infected MDBK cells with fluorescence microscopy at 72 hpi. The results showed that theaflavin effectively inhibited viral replication within 1 h of administration, and achieved a 50% inhibitory effect within 8 h of administration (Figure 7B). In the viral replication cycle, the longer the duration of exposure to the drug, the better the inhibitory effect, and the early addition of the drug was most likely to inhibit viral replication. Therefore, we suggest that theaflavin affects the initial post-entry stage and subsequent viral replication of LSDV.

Overall, these results demonstrate that theaflavin inhibits LSDV replication in the early stage of viral entry into cells.

## Discussion

As a orthopoxvirus, LSDV causes severe skin and visceral nodules in cattle, and poses a huge threat to the world’s cattle industry. Current control methods for LSD rely mainly on isolating and culling infected cattle in affected areas. However, the spread of LSDV is often incompletely blocked, and cattle farmers eagerly anticipate the emergence of effective and safe vaccines and antiviral drugs that target LSDV [19]. To develop a reliable LSDV research tool, we successfully used homologous recombination to construct a recombinant LSDV carrying the EGFP gen. Using standard virus purification procedures, we obtained the pure recombinant virus LSDV-ΔTK/EGFP. LSDV-ΔTK/EGFP displayed replication efficiency in MDBK cells indistinguishable from that of LSDV-WT, indicating that the insertion of EGFP into the TK gene did not affect the growth performance of the virus. The expression level of the EFGP gene in LSDV-ΔTK/EGFP-infected cells was closely related to viral replication, and we inferred that the growth of the reporter virus could be directly monitored with the EGFP signal. LSDV-ΔTK/EGFP was stably passaged in MDBK cells and various cell lines from different sources displayed different sensitivities to the recombinant virus. LSDV-WT infection confirmed this conclusion. We also preliminarily screened a compound library of traditional Chinese medicinal monomers for LSDV-directed antiviral drugs and found that emodin, aloe emodin, and theaflavin exerted strong inhibitory effects on LSDV. We confirmed the feasibility of using LSDV-ΔTK/EGFP for rapid antiviral screening experiments using fluorescence microscopy, and found that theaflavin mainly affects the replication stage of LSDV after it enters cells.

In this study, EGFP was first inserted into the TK gene with homologous recombination, and LSDV-ΔTK/EGFP was successfully rescued with multiple rounds of viral plaque purification. An ultrastructural analysis and growth curves indicated that there was no significant difference between the recombinant and WT viruses, indicating that the TK gene does not affect the growth of the virus. Another study has shown that deleting the TK gene does not affect the replication of goatpox virus (GTPV) and that TK is not essential for viral growth [20], consistent with our findings. In the virus sensitivity test, LSDV successfully infected various animal kidney cells, among which Vero (monkey), CRFK (cat), and MDCK (dog) cells showed the highest infection efficiency, whereas PK15 (pig) cells had the lowest infection efficiency. Interestingly, compared with the other four types of cells, LSDV was most likely to form typical lesions (cell aggregation, high refractive index, and nodule formation) in MDBK cells. Research has shown that cowpox virus infects various mammals, including humans, dogs, and cats, as does LSDV [21, 22]. LSDV is a member of the genus *Capripox* and has a strong host barrier. These characteristics suggest that LSDV is a potentially highly safe carrier system for widespread drug delivery and carrier vaccine development.

With the high-throughput screening of traditional Chinese medicinal monomers, we found that emodin, aloe emodin, theaflavin, 4-ethylphenol, and tulipalin exert good inhibitory effects on LSDV. Of these, emodin, aloe emodin, and theaflavin had the best inhibitory effects and greatest safety. Emodin and aloe emodin inhibited more than 10 viral infections in vitro and in vivo, including herpes simplex virus (HSV-1) [23, 24], African swine fever (ASFV) [25], human cytomegalovirus [26], coxsackie virus B [27, 28], enterovirus 71 (EV71) [29, 30], and influenza A virus [31, 32], with broad spectrum antiviral properties. Our study found that emodin and aloe emodin inhibit the replication of LSDV with IC_50_s of 10.86 μM and 10.13 μM, respectively. Studies have reported that emodin inhibits the replication of Japanese encephalitis virus and EV71 by upregulating the expression of interferon (IFN)-stimulated genes [29], whereas aloe emodin inhibits the replication of ASFV by inhibiting the NF-κB signal pathway, thus promoting cell apoptosis [33]. However, further research is required to clarify the mechanism by which these drugs inhibit LSDV replication. Theaflavin is mainly derived from black and yellow tea and inhibits the replication of various viruses, such as the novel Zika virus (ZIKV) [34], influenza A virus [35], HSV-1 [36], SARS-COV-2 [37], ASFV [38], feline calicivirus [39]. We found that theaflavin strongly inhibits LSDV replication, with an IC50 of 10.05 μM. Other studies have reported that theaflavin inhibits ASFV replication by disrupting cell lipid metabolism by activating the AMPK signaling pathway in cells in vitro. It has also been reported that theaflavin inhibits the replication of ZIKV by inhibiting the NSP5 MTase activity of the virus, with an IC50 of 10.10 μM [34]. In the present study, we found that theaflavin mainly obstructs LSDV by inhibiting viral entry into cells and its subsequent replication stages, consistent with its effects on ASFV and ZIKV. LSDV-WT infection was then used to evaluate drug inhibition test with a TCID_50_-based method. The results also showed that emodin, aloe emodin, and theaflavin had the best inhibitory effects, with IC50 values of 9.78 μM. 8.07 μM, and 11.5 μM, respectively, similar to those against LSDV-ΔTK/EGFP. However, the higher IC50 value for theaflavin may be attributable to errors in observing the viral cytopathic effect (CPE) with the naked eye, resulting in some cells infected with viral particles not being counted. Anemoside B4 (AB4) is a natural saponin component isolated from the roots of Chinese bulbul, which has various biological activities, including antitumor, immune modification, and immune adjuvant activities. Studies have shown that AB4 inhibits the proliferation of mouse intestinal viruses by regulating the type I IFN response [40]. However, AB4 also inhibits the secretion of cytokines in endothelial cells induced by porcine circovirus and in rat intestinal microvascular endothelial cells induced by lipopolysaccharide. Our results show that AB4 exerts an antiviral effect on LSDV. AB4 requires a larger dose to inhibit LSDV replication than several other drugs, with an IC50 of 845.65 μM. This does not favor its development as an antiviral drug, because high drug concentrations increase the costs. Among the six drugs tested, emodin, aloe emodin, theaflavin, and AB4 showed the lowest cytotoxicity, whereas ethylphenol and tulipalin were more cytotoxic and therefore unsuitable for drug development.

In this study, theaflavin inhibited the entry of LSDV into the post-entry stage and its replication. In the proliferation cycle of LSDV, viral particles enter the cell 1 h after they adsorb to the cell, and the complete genome is then released. When theaflavin was added to LSDV 1 h after adsorption, LSDV infection was maximally inhibited. Therefore, theaflavin may affect the post-entry stage of infection and the subsequent viral replication of LSDV. As a natural compound isolated from black tea, theaflavin has good anti-inflammatory and antiviral effects. Studies have shown that theaflavin reduces the accumulation of total cholesterol and triglycerides in cells, thereby inhibiting the replication of ASFV. However, the mechanism by which theaflavin inhibits LSDV replication is unclear and further research is required.

We established a recombinant transfer vector system for LSDV and rescued the reporter virus LSDV-ΔTK/EGFP. The reporter virus showed good stability in MDBK cells and different infectivities in various cell lines. The antiviral characteristics of the developed reporter virus were confirmed and we identified several traditional Chinese medicinal monomers with high anti-LSDV activity. The good inhibitory activity of theaflavin against LSDV infection in vitro was characterized. Our findings may prompt new ideas and the development of novel methods for the prevention and treatment of LSD, which should be particularly useful given the current lack of any treatment for this disease.

### Supplementary Information

**Additional file 1.** **A library of natural Chinese medicinal monomers (100 compounds) for antiviral compounds.**

## Data Availability

The data that support the findings of this study are available from the corresponding author upon reasonable request.
